# Broad specificity microbial enzymes: An annotated selection of World Wide Web sites relevant to the topics in microbial biotechnology

**DOI:** 10.1111/1751-7915.12270

**Published:** 2015-01-28

**Authors:** Lawrence P Wackett

## Broad specificity peroxiredoxin

### http://www.pnas.org/content/107/14/6240.full.pdf

This paper describes a broad specificity enzyme from *Treponema pallidum* that is involved in the detoxification of organic hydroperoxides.

## Broad specificity microbial N-demethylase

### http://mic.sgmjournals.org/content/157/2/583.full.pdf

This study describes the general characterization of an enzyme that catalyzes oxidative demethylation of N-methylated purines.

## Enzyme annotations: Hydrolases

### http://www.enzyme-database.org/downloads/ec3.pdf

This file contains a large listing of hydrolases with annotations on the general substrate ranges of the enzymes, some of which have a very wide substrate spectrum.

## Cyclohexanone monooxygenase from *Acinetobacter* NCIB 9871

### https://gmwgroup.harvard.edu/pubs/pdf/248.pdf

Cyclohexanone monooxygenase is a flavoprotein that catalyzes a Baeyer-Villiger type oxygenation reaction with cyclic ketones but also catalyzes a range of other reactions with distinctly different substrates.

## Review on enzyme promiscuity and opportunities for engineering

### http://www.weizmann.ac.il/Biological_Chemistry/scientist/Tawfik/papers/(109)BarEven_CoOpBiot_2013.pdf

This review article discusses a number of enzymes with broad specificity in the context of enzyme promiscuity.

## Soluble methane monooxygenase: KEGG database

### http://www.genome.jp/dbget-bin/www_bget?ec:1.14.13.25

Soluble methane monooxygenase accepts a large range of aliphatic, cyclic, and aromatic hydrocarbons and substituted derivatives of those hydrocarbons.

## Toluene dioxygenase reactions

### http://eawag-bbd.ethz.ch/tol/tdo.html

This web page contains a list of substrates for toluene dioxygenase (over 100) with links to further information about the reactions. It also contains references to the original literature describing the reactions.

## Naphthalene dioxygenase reactions

### http://eawag-bbd.ethz.ch/naph/ndo.html

This web page contains a list of substrates for naphthalene dioxygenase with links to further information about the reactions. It also contains references to the original literature describing the reactions.

## Naphthalene dioxygenase reactions updated

### http://www.ncbi.nlm.nih.gov/pubmed/24907321

A recent study broadened the substrate range of naphthalene dioxygenase to over 150 substrates. A large table is contained within the supplemental data to the paper.

## Broad specificity beta-lactamase

### http://www.ncbi.nlm.nih.gov/pmc/articles/PMC3633820/

This paper describes an enzyme that hydrolyzes and detoxifies a wide range of beta-lactamase antibiotics, which poses a serious concern with respect to the spread of antibiotic resistance.

## Use of promiscuous enzymes for organic synthesis: Thesis

### http://docserv.uni-duesseldorf.de/servlets/DerivateServlet/Derivate-29631/Kulig%20Justyna_PhD%20thesis_2013_v4.pdf

This thesis describes a range of broad specificity enzymes which can have utility in devising synthetic schemes for biotechnology.

## Substrate index: Sigma-Aldrich

### http://www.sigmaaldrich.com/life-science/metabolomics/enzyme-explorer/substrate-index.html

This listing is of substrates and it indicates the enzymes acting upon those substrates. Many of the substrates are useful in convenient enzyme assays for the substrates.

## Cytochrome P450: Protein databank

### http://www.rcsb.org/pdb/101/motm.do?momID=82

Cytochrome P450 monooxygenases are generally broad specificity catalysts. This page contains links to a number of structures of prominent members of this enzyme class.

## Substrate specificity of haloalkane dehalogenases

### http://www.biochemj.org/bj/435/0345/bj4350345.htm

Haloalkane dehalogenases catalyze the hydrolytic dehalogenation of aliphatic and cyclic haloalkanes. They generally have a fairly wide substrate range and this study compares them in this respect.

## Substrates of (S)-2-haloacid dehalogenase

### http://www.brenda-enzymes.org/enzyme.php?ecno=3.8.1.2#SUBSTRATE

Haloacid dehalogenases act on a range of halogenated aliphatic acids.

## *Candida antarctica* B lipase: BRENDA

### http://www.brenda-enzymes.info/enzyme.php?ecno=3.1.1.3&Suchword=&organism%5B%5D=Candida+antarctica&show_tm=0

*Candida antarctica* B lipase, sometimes known as CalB, has found great utility in selective hydrolysis and transesterification reactions. It is widely used industrially.

